# Investigation of proteome changes in osteoclastogenesis in low serum culture system using quantitative proteomics

**DOI:** 10.1186/s12953-016-0097-6

**Published:** 2016-03-31

**Authors:** Qi Xiong, Lihai Zhang, Shaohua Zhan, Wei Ge, Peifu Tang

**Affiliations:** Department of Orthopedics, General Hospital of Chinese PLA, Fuxing Road 28#, Haidian District, Beijing, 100853 China; National Key Laboratory of Medical Molecular Biology & Department of Immunology, Institute of Basic Medical Sciences, Chinese Academy of Medical Sciences, DongdanSantiao 5#, Dongcheng District, Beijing, 100005 China

**Keywords:** Osteoclast, Cell culture, Proteomics, Bioinformatics

## Abstract

**Background:**

RAW 264.7 cells can differentiate into osteoclasts when cultured in medium supplemented with 1 % FBS. However, the proteomic changes in the development of RAW 264.7 cells into osteoclasts in low serum culture system have not been elucidated. Therefore, we conducted quantitative proteomics analysis to investigate proteomic changes during osteoclastogenesis in low serum culture system.

**Results:**

Our study confirmed that mature multinucleated osteoclasts were generated in a low serum culture system, validated by upregulated expression of 15 characteristic marker proteins, including TRAP, CTSK, MMP9, V-ATPase and ITGAV. Proteomics results demonstrated that 549 proteins expressed differentially in osteoclastogenesis in low serum culture system. In-depth bioinformatics analysis suggested that the differentially expressed proteins were mainly involved in mitochondrial activities and energy metabolism, including the electron transport chain pathway, TCA cycle pathway, mitochondrial LC-fatty acid beta-oxidation pathway and fatty acid biosynthesis pathway. The data have been deposited to the ProteomeXchange with identifier PXD001935.

**Conclusion:**

Osteoclast formation is an ATP consuming procedure, whether occurring in a low serum culture system or a conventional culture system. In contrast to osteoclasts formed in conventional culture system, the fatty acid biosynthesis pathway was upregulated in osteoclasts cultured in low serum condition.

**Electronic supplementary material:**

The online version of this article (doi:10.1186/s12953-016-0097-6) contains supplementary material, which is available to authorized users.

## Background

Osteoporosis is a skeletal disorder characterized by diminished bone mineral density and deteriorated bone micro-structure, which consequently leads to increased fracture risk, especially in the elder population. Epidemiological study revealed that more than 75 million people suffer from osteoporosis in United States, Europe, and Japan [1]. It is estimated that approximately 40 % of Caucasian women and 13 % of Caucasian men aged over 50 years old will suffer from osteoporosis-related fracture in the United States [2, 3]. Osteoporosis and osteoporosis-related fractures therefore have become a major public health concern, and impose enormous health care costs. Burge et al. demonstrated that annual costs for osteoporosis-related fractures were US$13.7 to US$20.3 billion, and predicted that the costs would increase to US$25.3 billion annually by 2025 in the United States [4]. Therefore, investigating the pathological mechanisms of osteoporosis will be of great help to reduce the tremendous osteoporosis related costs and promote life quality of elder populations.

Over-activation of bone resorption plays a critical role in the pathological mechanisms of osteoporosis [5]. To date, large multinucleated cells termed osteoclasts are the exclusive cells known to have the ability of bone resorption. Thus, studying the molecular mechanisms of osteoclast formation is essential for investigating pathological mechanisms of osteoporosis. Osteoclasts derive from monocyte/macrophage cells [6]. Followed activation of the receptor activator of nuclear factor B ligand (RANKL)/RANK signaling pathway by RANKL, downstream signaling cascades are stimulated, including the IκB kinase (IKK) signaling, c-Jun N-terminal kinase (JNK) signaling, NF-κB signaling and MAPK signaling [7–10]. Subsequently, monocyte/macrophage cells differentiate and fuse into mature multinucleated osteoclasts, regulated by nuclear factor of activated T-cells, cytoplasmic, calcineurin-dependent 1 (NFATc1) and microphthalmia-associated transcription factor (MITF) [11]. However, the molecular mechanisms of osteoclast formation still need to be elucidated.

The cultivation of osteoclasts in vitro is a prerequisite for studying the molecular mechanisms of osteoclasts. Traditionally, a 10 % volume fraction of serum is used to culture osteoclasts. We and other researchers previously demonstrated that RAW264.7 cells, an osteoclast precursor cell line, could differentiate into mature bone resorbing osteoclasts when cultured both in 1 % serum and serum-deprived medium [12, 13]. Our previous study revealed that a number of proteins and molecular signaling pathways, including electron transport chain and oxidative phosphorylation pathways, were altered in RAW264.7 cells cultured in low serum compared to those cultured in the conventional 10 % culture system [13]. An et al. have performed proteomics analysis to study the changes in global protein expression during osteoclastogenesis in conventional culture system [14]. However, to the best of our knowledge, the proteomic changes that occur in RAW 264.7 cells as they differentiate into osteoclasts in a low serum culture system has not been reported. In the present study, we used TMT labeling to analyze the quantitative proteomic changes during osteoclastogenesis in medium supplemented with 1 % FBS.

## Results

### Conformation of osteoclastogenesis in a low serum culture system

**Fig. 1:**
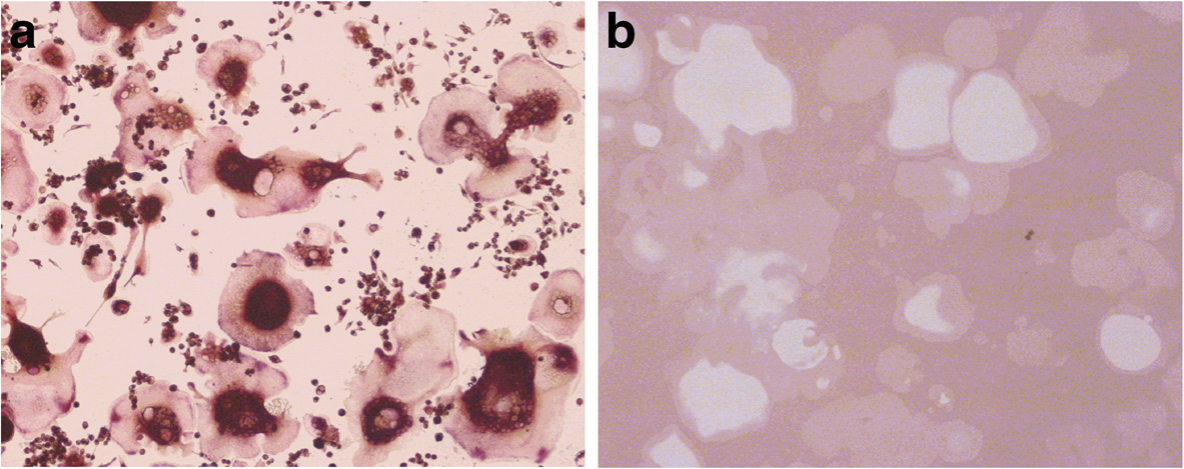
Confirmation of osteoclast formation in low serum culture system using TRAP staining and bone resorption activity test. **a** TRAP-positive multinucleated osteoclasts were successfully obtained. **b** Bone mineral surface was resorbed remarkably by osteoclasts generated in low serum culture system

To validate the formation of osteoclasts in medium supplemented with 1 % FBS, we performed TRAP staining and bone resorption assay. After 5 days cultivation, TRAP-positive cells with more than 3 nuclei could be observed (Fig. 1a), and the simulated bone mineral surface was remarkably resorbed (Fig. 1b). These results confirmed that formation of mature multinucleated osteoclasts could be obtained in a low serum culture system. The tartrate-resistant acid phosphatase type 5 (TRAP or ACP5; Accession Number: Q05117), cathepsin K (CTSK; Accession Number: P55097), matrix metalloproteinase-9 (MMP9; Accession Number: P41245) are characteristic osteoclast marker proteins [15]. To ensure that the large multinucleated cells generated in our study were osteoclasts, quantitative proteomic data was analyzed. Our results revealed that the three characteristic marker proteins were significantly upregulated in these differentiated cells. Compared to RAW 264.7 cells, TRAP, CTSK and MMP9 in multinucleated cells were upregulated 2.59, 5.21 and 2.82-fold respectively (Table 1). The vacuolar-type H^+^-ATPase (V-ATPase) proton pump complex is typically located on the ruffled border plasma membrane of osteoclasts [16], and can also be used as biomarker for mature osteoclasts. Our results identified 11 subunits of V-ATPase; all of these subunits were significantly upregulated (Table 1). Furthermore, bone resorbing osteoclasts require integrin alpha-V (ITGAV; Accession Number: P43406) for the formation of the sealing zone between osteoclasts and bone surface [17]. Our results showed that the ITGAV was upregulated 2.53-fold (Table 1). These findings clearly suggest that the giant multinucleated cells formed in low serum culture system were osteoclasts.

**Table 1:** Quantitative proteomics data of characteristic biomarkers for osteoclasts

Accession	Description	Score	Unique peptide	Tubulin adjust (128/127)
P55097	Cathepsin K OS = Mus musculus GN = Ctsk	168.11	7	5.21
P41245	Matrix metalloproteinase-9 OS = Mus musculus GN = Mmp9	58.12	15	2.82
Q05117	Tartrate-resistant acid phosphatase type 5 OS = Mus musculus GN = Acp5	104.03	10	2.59
Q9R1Q9	V-type proton ATPase subunit S1 OS = Mus musculus GN = Atp6ap1	25.62	5	2.50
Q8BVE3	V-type proton ATPase subunit H OS = Mus musculus GN = Atp6v1h	160.48	19	2.37
Q9CR51	V-type proton ATPase subunit G 1 OS = Mus musculus GN = Atp6v1g1	76.40	4	3.35
Q9D1K2	V-type proton ATPase subunit F OS = Mus musculus GN = Atp6v1f	67.21	9	2.45
P50518	V-type proton ATPase subunit E 1 OS = Mus musculus GN = Atp6v1e1	219.66	16	2.59
P57746	V-type proton ATPase subunit D OS = Mus musculus GN = Atp6v1d	130.06	12	2.33
Q80SY3	V-type proton ATPase subunit d 2 OS = Mus musculus GN = Atp6v0d2	134.85	13	3.46
P51863	V-type proton ATPase subunit d 1 OS = Mus musculus GN = Atp6v0d1	71.67	13	2.54
Q9Z1G3	V-type proton ATPase subunit C 1 OS = Mus musculus GN = Atp6v1c1	216.86	24	2.62
P62814	V-type proton ATPase subunit B, brain isoform OS = Mus musculus GN = Atp6v1b2	762.21	36	2.16
P50516	V-type proton ATPase catalytic subunit A OS = Mus musculus GN = Atp6v1a	658.00	38	2.12
P43406	Integrin alpha-V OS = Mus musculus GN = Itgav	17.86	4	2.53

### Western blot analysis of the differentially expressed proteins

**Fig. 2:**
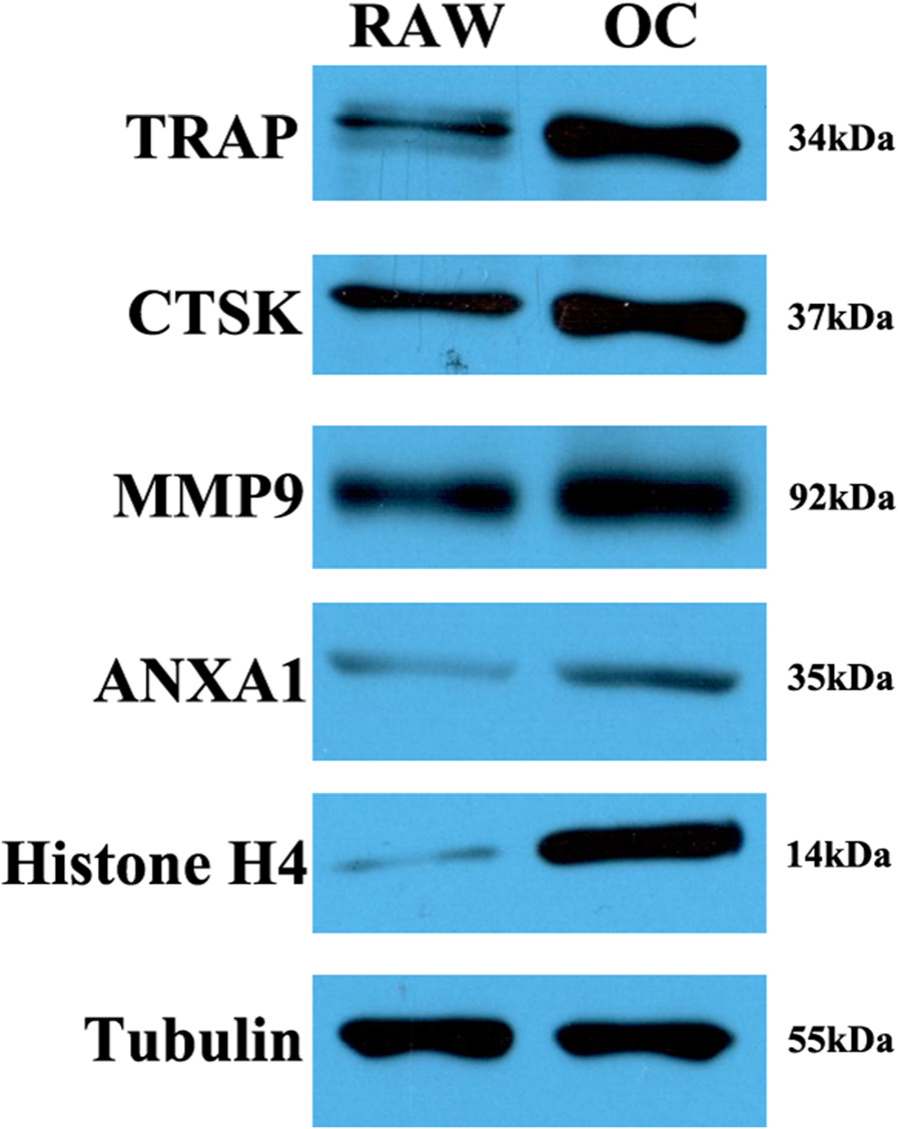
Validation of the LC-MS/MS data and osteoclast formation by western blot analysis. Characteristic biomarkers of osteoclasts were seen. TRAP, CTSK, and MMP-9 were significantly upregulated in osteoclasts. Similar to the results from LC-MS/MS, ANXA1 and histone H4 were upregulated in osteoclasts compared to RAW 264.7 cells

To validate the formation of osteoclasts and the results of the quantitative LC-MS/MS data, we performed western blot analysis of three characteristic proteins of osteoclasts (TRAP, CTSK, MMP9) and two other differentially expressed proteins (ANXA1, Histone H4). The expression of the three characteristic osteoclast proteins was significant upregulated on western blots, confirming the formation of osteoclasts in the low serum system. In addition, the expression of ANXA1 and histone H4 were also upregulated, as showed in our quantitative LC-MS/MS data. These findings indicated that our LC-MS/MS data and subsequent analysis were confirmed by western blotting, suggesting that the analysis results accurately reflect changes in protein expression (Fig. 2).

### Quantitative analysis of proteomic changes during osteoclastogenesis in a low serum system

We performed quantitative proteomic analysis to compare the global protein expression of RAW 264.7 cells with those of the osteoclasts. Our results identified a total of 6567 proteins, of which 4656 proteins with a score of 10 or more and 2 or more unique peptides identified were selected for further analysis. Of the 4656 proteins with high confidence, 549 proteins expressed differentially when RAW 264.7 cells differentiated into osteoclasts. Among the 549 differentially expressed proteins, 98.5 % (541/549) were significantly upregulated, while only 1.5 % (8/549) of proteins were downregulated (Additional file 1: Table S1). These results indicated that a great many proteins were upregulated, and suggested that RAW 264.7 cells were highly activated in the development into osteoclasts in the low serum culture system.

### Identification and classification of the differentially expressed proteins

**Fig. 3:**
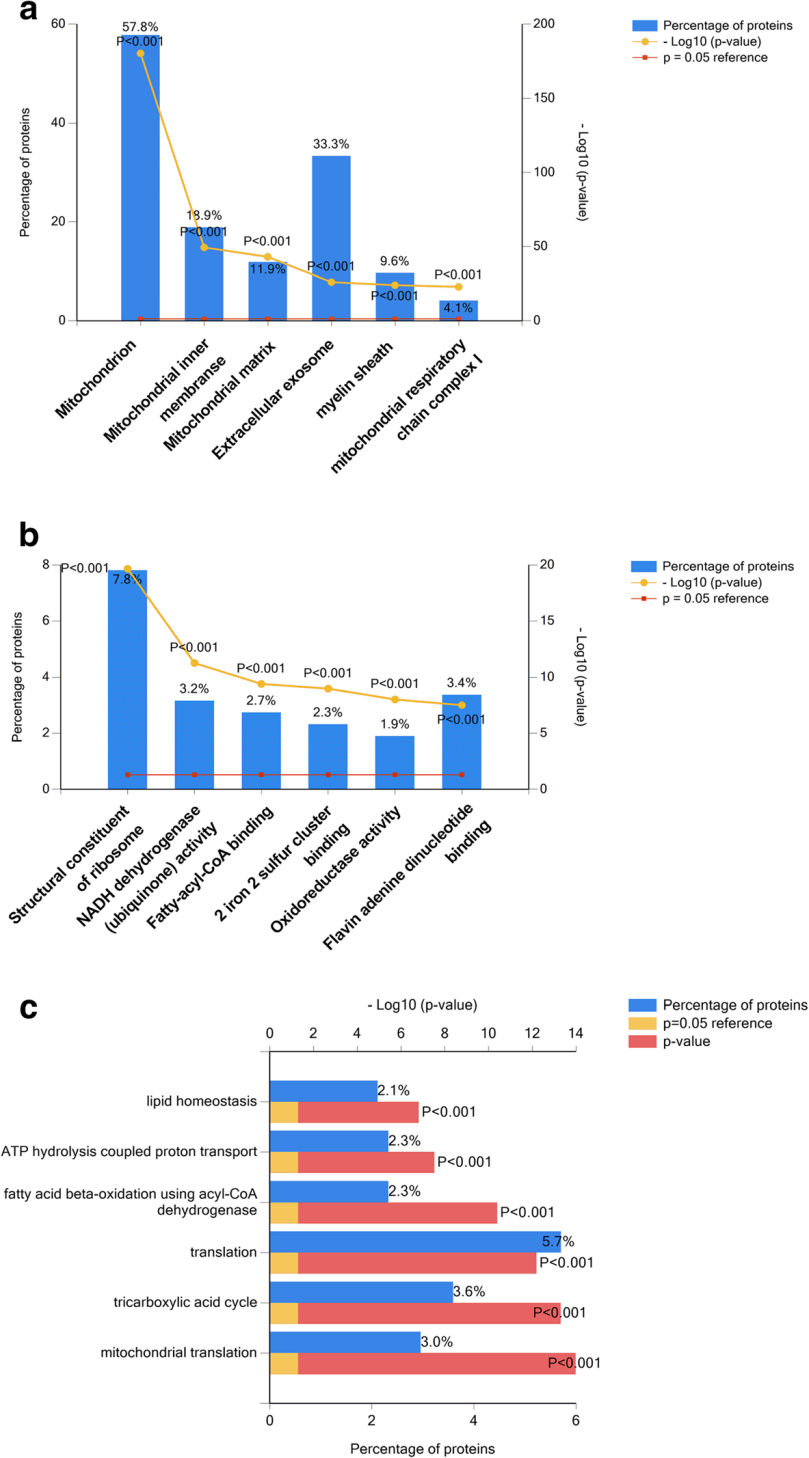
Identification and classification of the upregulated proteins. **a** Column graph of cellular component enriched in upregulated proteins. **b** Column graph of molecular function enriched in upregulated proteins. **c** Bar graph of biological process enriched in upregulated proteins

**Fig. 4:**
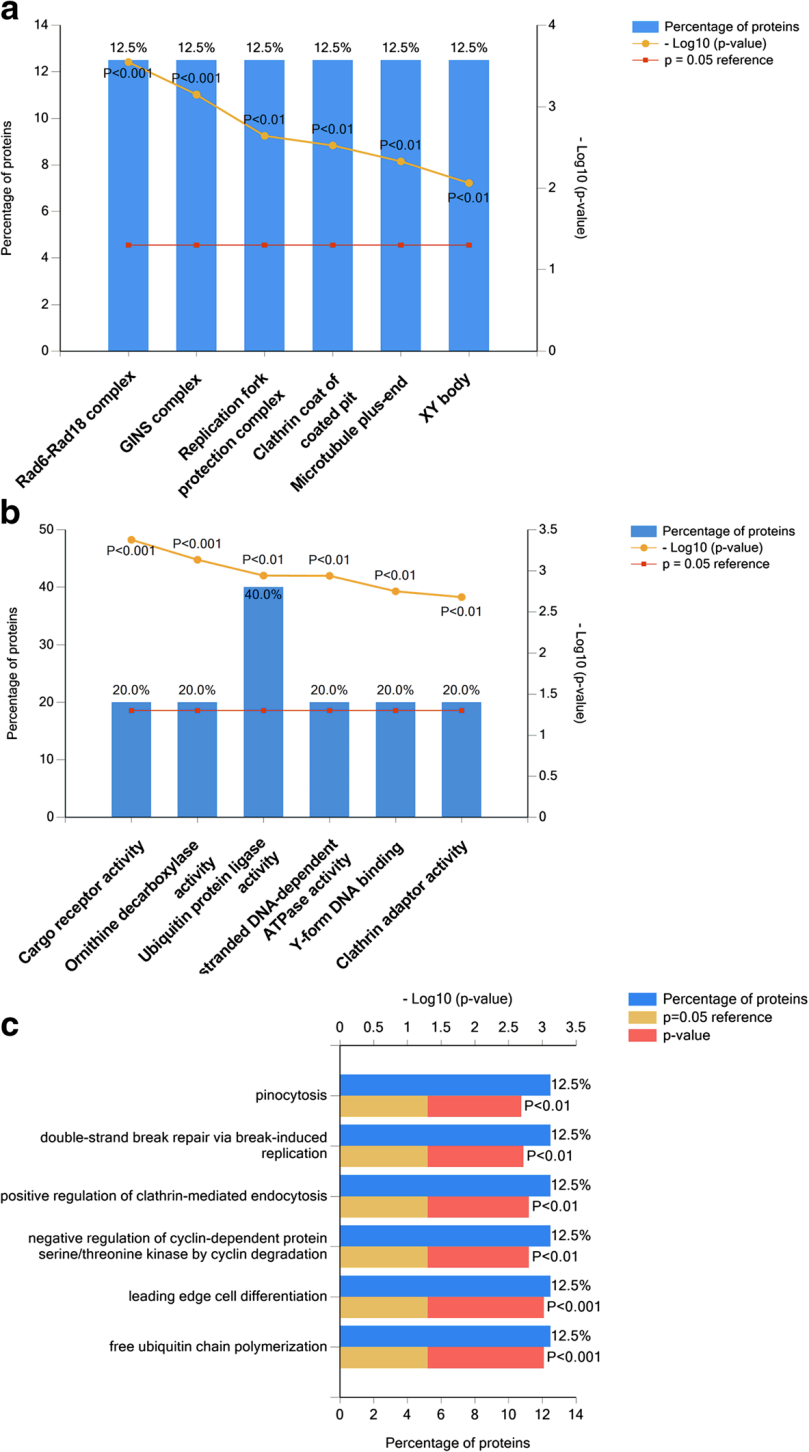
Identification and classification of the downregulated proteins. **a** Column graph of cellular component enriched in downregulated proteins. **b** Column graph of molecular function enriched in downregulated proteins. **c** Bar graph of biological process enriched in downregulated proteins

To understand the specific proteomic changes, we performed enrichment analysis using FunRich software to identify and classify the differentially expressed proteins. Of the 549 upregulated proteins, 548 proteins were successfully mapped to the database. In the context of cellular component, 540 of the 541 upregulated proteins and 8 downregulated proteins matched with the database. Upregulated proteins were mostly enriched in mitochondria, mitochondrial inner membrane, mitochondrial matrix, extracellular exosome, myelin sheath and mitochondrial respiratory chain complex I (Fig. 3a). Downregulated proteins were enriched in the Rad6-Rad18 complex, GINS complex, replication fork protection complex, clathrin coat of coated pit, microtubule plus-end and XY body (Fig. 4a). These findings indicated that proteomic changes mainly occurred in mitochondria when RAW 264.7 cells differentiated into osteoclasts in the low serum culture system.

According to the molecular function analysis, 482 upregulated proteins and 7 downregulated proteins matched with the database. Our results revealed that upregulated proteins were mainly enriched in molecular functions of structural constituent of ribosome, NADH dehydrogenase (ubiquinone) activity, fatty-acyl-CoA binding, 2 iron 2 sulfur cluster binding, oxidoreductase activity and Flavin adenine dinucleotide binding (Fig. 3b). Downregulated proteins were enriched in cargo receptor activity, ornithine decarboxylase activity, ubiquitin protein ligase activity, single-stranded DNA-dependent ATPase activity, Y-form DNA binding, and clathrin adaptor activity and etc. (Fig. 4b).

In the context of biological process, upregulated proteins were mainly enriched in lipid homeostasis, ATP hydrolysis coupled proton transport, fatty acid beta-oxidation using acyl-CoA dehydrogenase, translation, tricarboxylic acid (TCA) cycle (also termed citric acid cycle) and mitochondrial translation (Fig. 3c). By contrast, downregulated proteins were mainly involved in biological processes of pinocytosis, double-strand break repair via break-induced replication (Fig. 4c). These findings indicated that the differentially expressed proteins were mainly involved in mitochondria and energy metabolism during osteoclastogenesis in the low serum culture system.

### Functional interaction network analysis of the differentially expressed proteins

**Fig. 5:**
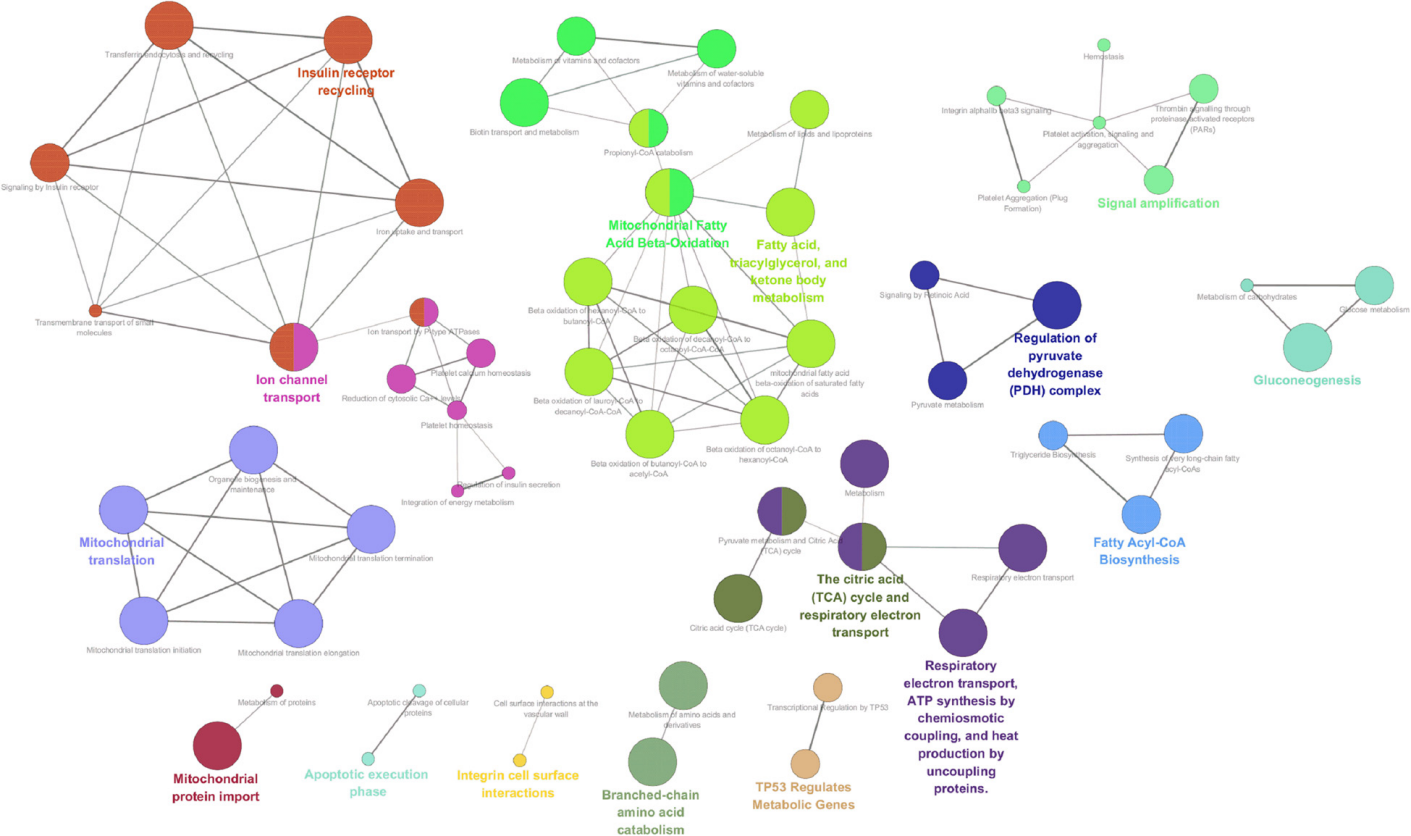
Functional interaction network analysis of the differentially expressed proteins mapped to the REACTOME database using ClueGO cytoscape plugin

To investigate functional interaction network of the differentially expressed proteins, the ClueGO Cytoscape plugin was used. Of the 549 differentially expressed proteins, 548 proteins were successfully mapped to the REACTOME database, and one protein (Accession Number: B9EJ86) failed to map to the database. Our results revealed that the differentially expressed proteins mainly participated in mitochondrial fatty acid beta-oxidation, mitochondrial translation, fatty acyl-CoA biosynthesis, the TCA cycle as well as respiratory election transport, among others (Fig. 5). These findings again indicated that the differentially expressed proteins were implicated in mitochondrial activities and energy metabolism activities.

### Molecular pathways analysis of the differentially expressed proteins

**Fig. 6:**
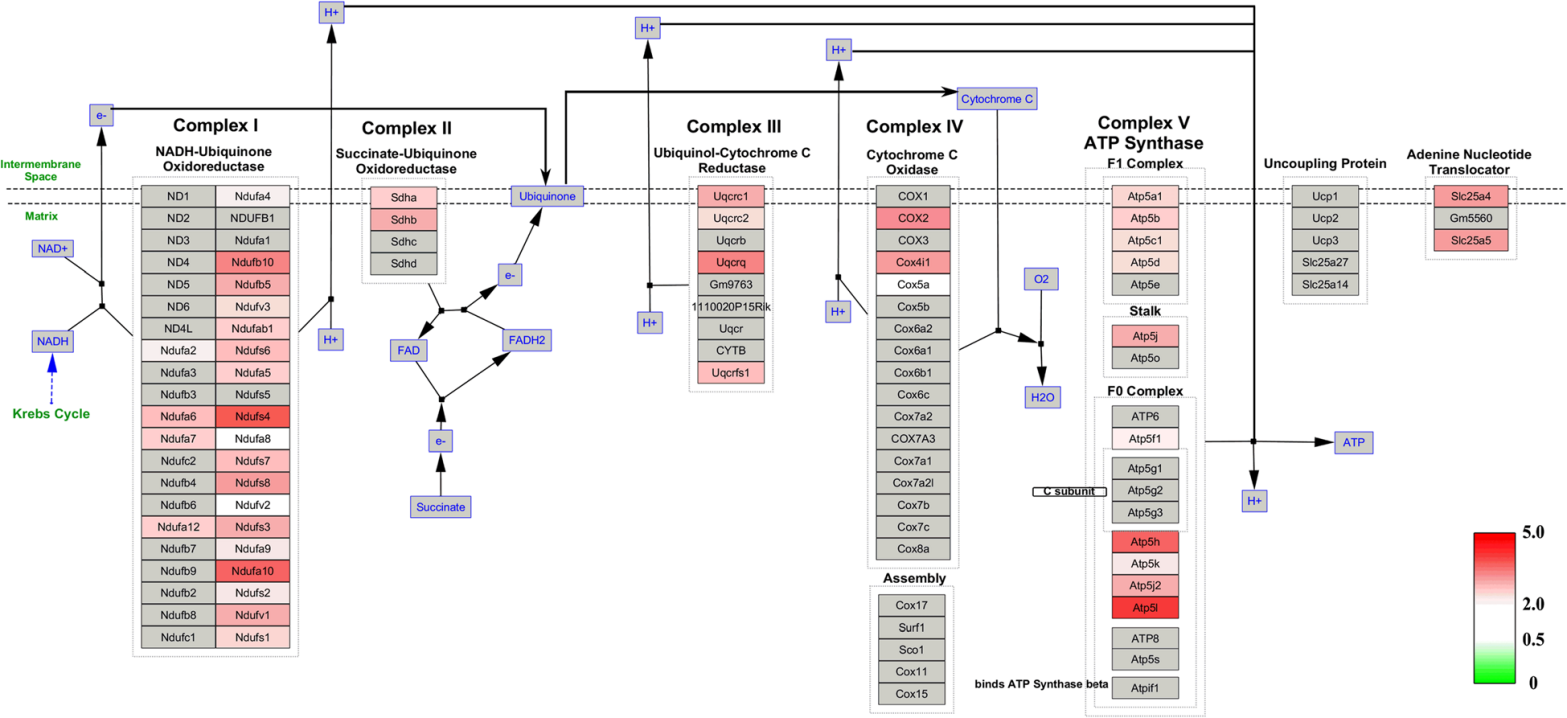
Visualization of all differentially expressed proteins mapped to the electron transport chain pathway in the development of RAW 264.7 cells into osteoclasts in low serum culture system. *Gray boxes* indicate proteins not mapped, *green boxes* indicate downregulated proteins, *red boxes* indicate upregulated proteins. Color intensity is adjusted to indicate the ratio value

**Fig. 7:**
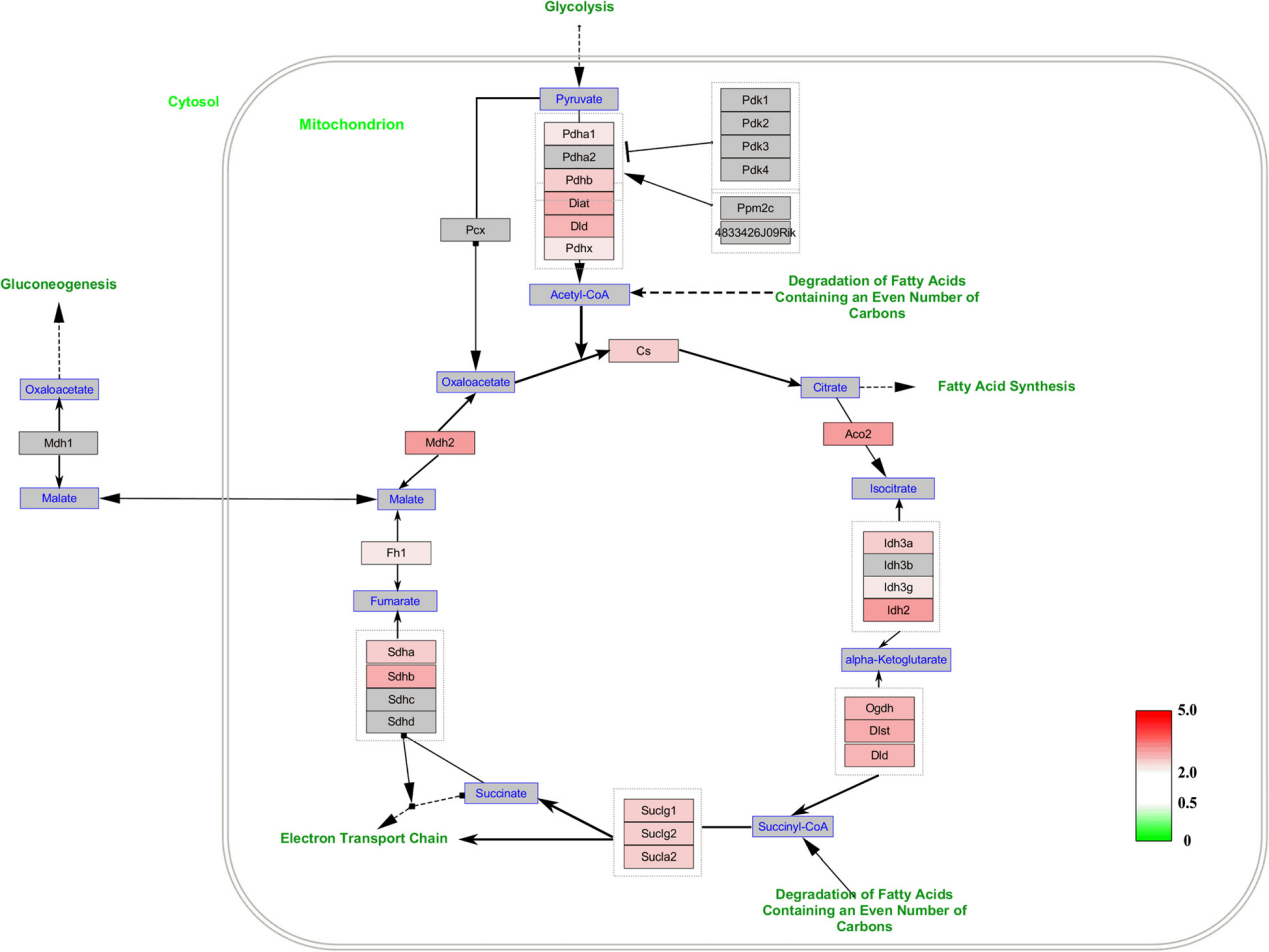
Visualization of all differentially expressed proteins mapped to the TCA cycle pathway in the development of RAW 264.7 cells into osteoclasts in low serum culture system. *Gray boxes* indicate proteins not mapped, *green boxes* indicate downregulated proteins, *red boxes* indicate upregulated proteins. Color intensity is adjusted to indicate the ratio value

**Fig. 8:**
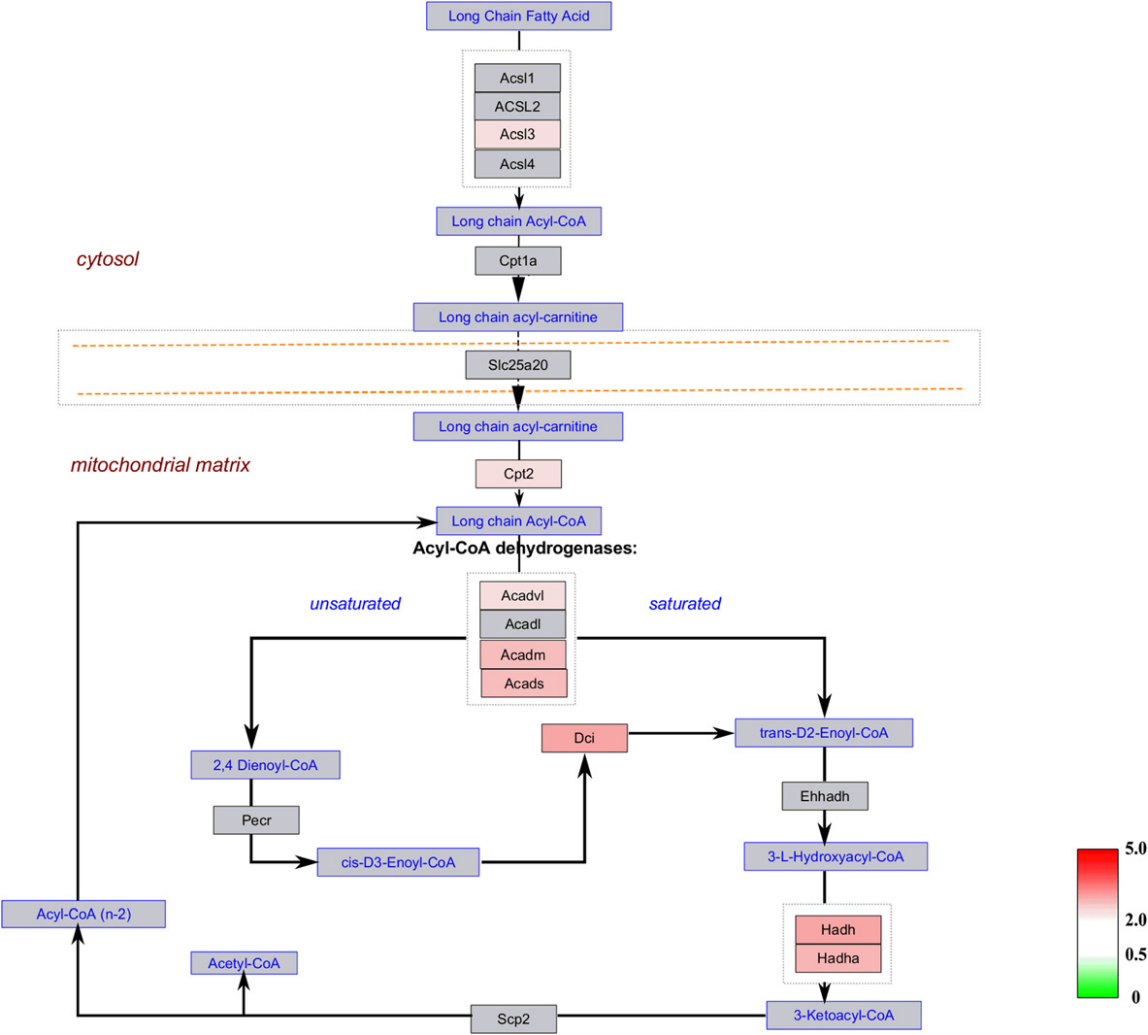
Visualization of all differentially expressed proteins mapped to the mitochondrial LC-fatty acid beta-oxidation pathway in the development of RAW 264.7 cells into osteoclasts in low serum culture system. *Gray boxes* indicate proteins not mapped, *green boxes* indicate downregulated proteins, *red boxes* indicate upregulated proteins. Color intensity is adjusted to indicate the ratio value

**Fig. 9:**
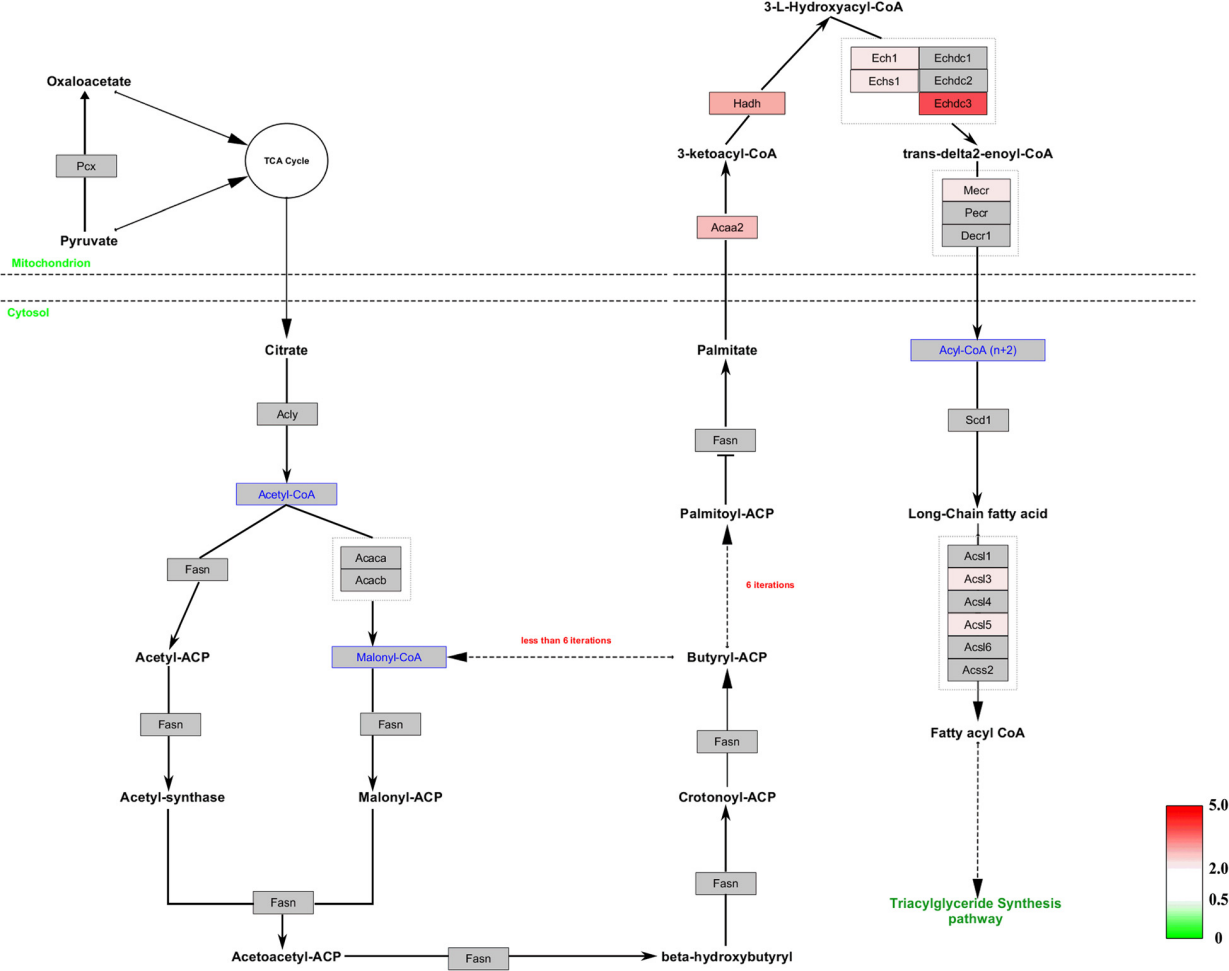
Visualization of all differentially expressed proteins mapped to the fatty acid biosynthesis pathway in the development of RAW 264.7 cells into osteoclasts in low serum culture system. *Gray boxes* indicate proteins not mapped, *green boxes* indicate downregulated proteins, *red boxes* indicate upregulated proteins. Color intensity is adjusted to indicate the ratio value

To further investigate the interaction networks of the differentially expressed proteins, we used WebGestalt online toolkit to perform pathway enrichment analysis based on the Wikipathways database, and used the Wikipathways Cytoscape plugin to visualize the changes in the molecular pathways. Consistent with functional interaction network analysis, molecular pathway enrichment analysis demonstrated that electron transport chain pathway, TCA cycle pathway, mitochondrial LC-fatty acid beta-oxidation pathway and fatty acid biosynthesis pathway were significantly altered (Additional file 2: Table S2). In the electron transport chain, expression of 45 matched proteins were upregulated; these proteins were involved in mitochondrial complex I (NADH-Ubiquinone oxidoreductase complex), the ubiquinol-cytochrome C reductase complex and ATP synthase F1 complex (Fig. 6). In the TCA cycle pathway, 19 identified proteins were upregulated (Fig. 7). In the mitochondrial LC-fatty acid beta-oxidation pathway, our results showed that seven proteins, including ACADS, ACADVL, HADHA, HADH, ACADM, CPT2 and ACSL3, were upregulated, especially those involved in saturated fatty acid (Fig. 8). Similarly, in the fatty acid biosynthesis pathway, all eight identified proteins were upregulated, especially in mitochondrion (Fig. 9). Together, our results suggested that mitochondrial activity and energy metabolism were remarkably upregulated in the differentiation of RAW 264.7 cells into osteoclasts in the low serum culture system.

## Discussion

In the present study, we investigated the proteomic changes during osteoclastogenesis in medium supplemented with 1 % FBS. Consistent with our previous study [13], our results confirmed that large TRAP-positive multinucleated osteoclasts with bone resorbing capacity were successfully obtained by this culturing procedure, validated by upregulation of 15 characteristic marker proteins, including TRAP, CTSK, MMP9, V-ATPase and ITGAV, three of which were also confirmed by western blot analysis. Previous study found 867 proteins (492 down-regulated proteins and 375 upregulated proteins) altered between osteoclasts and RAW264.7 cells [14], while our study found 549 proteins (541 upregulated proteins and 8 downregulated proteins) expressed differentially during osteoclastogenesis in the low serum culture system, and almost all the differentially expressed proteins were significantly upregulated. Integrated bioinformatics analysis indicated that these differentially expressed proteins were mainly involved in mitochondrial activities and energy metabolism, including the electron transport chain pathway, TCA cycle pathway, mitochondrial LC-fatty acid beta-oxidation pathway and fatty acid biosynthesis pathway.

The electron transport chain is a complex biological process that transfers electrons from electron donors to electron acceptors [18]. The electron transport chain pathway is responsible for the synthesis of ATP, the most commonly consumed chemical energy utilized in a diversity array of cellular biological activities, including osteoclast formation [14, 19-21]. In eukaryotic cells, ATP is mainly generated in mitochondria. An et al. found that mitochondrial changes were critical in osteoclastogenesis in the conventional 10 % serum culture system [14]. Moreover, our previous study indicated that the expression of the electron transported chain in RAW264.7 cultured in low serum system was downregulated [13]. In this study, our results showed that the differentially expressed proteins were mainly located in mitochondria, suggesting changes in electron transported chain in osteoclasts formed in the low serum culture system. Similar to our results, Morten et al. found increased mitochondrial electron transport chain activity in the differentiation of human CD14 positive monocytes differentiating into osteoclasts under hypoxia conditions [22]. Moreover, Jin et al. reported that a mitochondrial complex 1 subunit Ndufs4 deletion caused systemic inflammation and osteopetrosis, and suggested that mitochondrial complex I promoted osteoclast differentiation, while inhibited macrophage activation [23]. In agreement with Jin et al., we found that mitochondrial complex I was activated in osteoclasts formed in low serum culture system. Taken together, these findings suggested that osteoclasts formation is an energy consuming procedure, regardless of the culture environmental condition.

ATP synthesis depends on the products of citric acid cycle (also termed TCA cycle) and fatty acid oxidation [24, 25]. Dodds et al. found that osteoclast formation was related to enhanced TCA cycle and increased fatty acid oxidation [26]. Our study supports this conclusion, as bioinformatics analysis showed differentially expressed proteins enriched in TCA cycle pathway and the mitochondrial LC-fatty acid beta-oxidation pathway. The TCA cycle is a biological process that oxidizes acetyl-CoA for ATP production [27]. Our results showed high activation of TCA cycle pathway in osteoclasts generated in medium supplemented with 1 % FBS. Fatty acids are carboxylic acids, which have a long aliphatic tail [28]. Fatty acid oxidation mostly occurs in the mitochondria and is an energy productive biological procedure. Adamek et al. suggested that fatty acid oxidation played an important role in the energy metabolism of bone cells, including osteoblast and fibroblasts [29]. However, the role of fatty acid oxidation in osteoclasts has not been reported. In this study, our results demonstrated that differentially expressed proteins from the fatty acid oxidation pathway upregulated significantly, indicating a role of fatty acid oxidation in the development of RAW 264.7 cells into osteoclasts in the low serum culture system. Again, these findings imply that formation and bone resorption of osteoclast require high ATP production.

Since both the TCA cycle and fatty acid oxidation are associated with fatty acid biosynthesis, we also analyzed alternations of fatty acid biosynthesis pathway in osteoclastogenesis in the low serum culture system. A previous study by Cornish et al. indicated that saturated fatty acid suppressed osteoclast formation in conventional culture system [30]. Moreover, Kim et al. suggested that a medium-chain fatty acid suppressed osteoclastogenesis by inhibiting RANKL-induced IκBα phosphorylation, p65 nuclear translocation, and NF-κB transcriptional activity. They also found that NFATc1 was inhibited by medium-chain fatty acid in osteoclastogenesis in conventional culture system [31]. In contrast, we found that fatty acid biosynthesis, mainly of saturated fatty acid, was upregulated in mitochondrion of osteoclasts formed in the low serum culture system. This difference may be because the osteoclasts were cultured in medium supplemented with low serum in our study. Therefore, our study suggested a different process of fatty acid biosynthesis in osteoclasts formed in low serum culture system from those in conventional culture system.

Additionally, cells are always pre-treated with serum free medium for 24 h or less before the start of the experiments to enhance the sensitivity of cells to drugs. We hypothesize that there might be differences between the proteomes of osteoclasts cultured in low serum system and a serum-free system for only 24 h. However, to the best of our knowledge, the effect of serum-free treatment on the osteoclast proteome has not been reported. Therefore, the comparison of effects of continuous low serum and transient serum free on osteoclasts cannot be done. Larsson et al. previously investigated the effects of short exposure to serum-free medium on 3 T3 cells proliferation cycle, and suggested that 3T3 cells are very sensitive to serum depletion during the first part of G1; only a short exposure to serum-free medium is sufficient for the cells to leave the cell cycle [32]. However, the effects of serum-free pretreatment on osteoclast differentiation has not been reported.

## Conclusion

In conclusion, our study confirmed that osteoclasts could be obtained in low serum culture system (1 % FBS), and demonstrated that osteoclast formation is an ATP consuming process, no matter if cells are cultured in a low serum culture system or a conventional culture system. Like osteoclast formation in the conventional culture system, the proteomic changes in osteoclastogenesis in the low serum culture system mainly involve mitochondrial activity and energy metabolism. Notably, our results showed that the fatty acid biosynthesis pathway was upregulated in osteoclasts cultured in low serum condition, the molecular mechanisms of which need further investigation.

## Methods

### RAW264.7 cell cultivation and osteoclastogenesis

RAW 264.7 cells (obtained from the Chinese Academy of Medical Sciences (Beijing, China)) were cultured at a density of 1.5 × 10^5^ cells/ml in 6 well plates. The cells were divided into two groups. The cells were cultured with or without 30 ng/ml RANKL (462-TEC-010, R&D Systems) in α-MEM (11095–080, Life Technologies) supplemented with 1 % (v/v) FBS (26140079, Life Technologies) at 37 °C in a 5 % CO_2_ incubator. The medium was refreshed every other day. Both sets of experiments were run in triplicated independently. On Day 5, RAW 264.7 cells and osteoclasts were harvested and pooled. Subsequently, the pooled cells were used for proteomic analysis.

### TRAP staining and bone resorption assay

TRAP staining and bone resorption assays were performed to confirm the formation of mature osteoclasts as described previously [33]. Mature osteoclasts were defined as TRAP-positive cells containing three or more nuclei. TRAP staining was conducted using the Acid Phosphatase Leukocyte kit (387–1, Sigma Aldrich) according to the manufacturer’s protocol. Briefly, cells were rinsed three times with cold PBS. Then, cells were fixed with Fixative Solution (Citrate Solution, acetone and 37 % formaldehyde) and rinsed thoroughly. Cells were incubated in staining solution for 1 h in a 37 °C water bath protected from light.

The Osteo Assay Surface Plate (3987, Corning) was used to evaluate the bone resorbing capacity of osteoclasts. On Day 5, following the aspiration of the medium, 100 μl of 10 % bleach solution was added to each well and incubated for 5 min. Toluidine blue staining was conducted to enhance the contrast for bone resorbing pit image analysis.

### Sample preparation and TMT labeling

Sample preparation and labeling were performed as described by Xiong et al. [34]. In brief, cells were washed three times with cold PBS, and then lysed with lysis buffer (8 M urea in PBS, 1 × cocktail, 1 mM PMSF). Cell lysates were centrifuged at 16,000 × g for 10 min at 4 °C, the supernatants were collected, and protein concentrations were measured with a Nanodrop2000. Following incubation in 10 mM dithiothreitol (17131801, GE Healthcare) at 50 °C for 1 h, 100 μg of protein was incubated with 25 mM indole acetic acid (RPN6302, GE Healthcare) in the dark for 2 h. Then, trypsin/Lys-C Mix (V5072, Promega) was used to digest proteins overnight at 37 °C at a protein/protease ratio of 25:1, and the reaction was quenched by heating at 60 °C. Proteins digests were desalted, dried and solved in 200 mM triethylammonium bicarbonate buffer. TMT Isobaric Label Reagent Set (90061, Thermo Scientific) was used to label proteins according to the manufacturer’s instructions. Proteins extracted from osteoclasts and RAW 264.7 cells were labeled with 0.8 mg TMT6-128 or TMT6-127, respectively. Equal amounts of the labeled protein digests from both groups were pooled, dried and solved in 0.1 % trifluoroacetic acid. Then the protein digests were finally solved in 100 μl of 0.1 % trifluoroacetic acid for mass spectrometry (MS) analysis.

### High-performance liquid chromatography (HPLC)

Fractionation of pooled protein digests was performed as described by van Ulsen et al. [35]. Briefly, the pooled TMT labeled protein digests were dissolved in 100 μl 0.1 % formic acid for HPLC analysis (UltiMate 3000 UHPLC, Thermo Scientific) using an Xbridge BEH300 C18 column (4.6 × 250 mm^2^, 5 μm, 300 Å, Waters). Fifty fractions were collected at 1.5 min intervals. The fractions were dried in a vacuum concentrator. Then, 20 μl 0.1 % FA was used to dissolve the fractions for LC-MS/MS analysis.

### LC-MS/MS analysis

LC-MS/MS was conducted using a Q Exactive mass spectrometer. The protein digests were separated using a 120 min gradient elution at a flow rate of 0.3 μl/min using the UltiMate 3000 RSLCano System (Thermo Scientific). A directly interfaced Q Exactive Hybrid Quadrupole-Orbitrap Mass Spectrometer (Thermo Scientific) was used to analyze the protein digests. We use a home-made fused silica capillary column (75 μm × 150 mm, Upchurch, Oak Harbor, WA, USA) packed with C18 resin (300 Å, 5 μm, Varian Lexington, MA, USA) as the analytical column. Xcalibur 2.1.2 software was used with the Q Exactive mass spectrometer in data-dependent acquisition mode. Ten data-dependent MS/MS scans at 27 % normalized collision energy were performed. Thereafter, a single full-scan mass spectrum in Orbitrap (400–1,800 m/z, 60, 000 resolution) was conducted.

### Western blot analysis

To validate the LC-MS/MS data and to confirm the formation of osteoclasts, western blot analysis of three characteristic osteoclast biomarkers and two other altered proteins was performed according to standard procedure with minor modifications. Equal amounts of total proteins of RAW264.7 cells and osteoclasts cultured in low serum (20 μg) were separated by SDS-PAGE on 12 % gel and transferred to nitrocellulose membranes. Membranes were blocked at room temperature for 1 h in Tris Buffer Saline with Tween 20 (TBST) with 5 % nonfat milk. Then, membranes were incubated with anti-TRAP (sc-28204), anti-CTSK (ab19027), anti-histone H4 (ab10158), anti-MMP-9 (ab137867), anti-ANXA1 (sc-12740) and anti-beta tubulin (internal control) antibody (KM9003) at 4 °C overnight. After washing in TBST for 15 min, membranes were incubated with goat anti-rabbit horseradish peroxidase (HRP)-conjugated IgG for 1 h at room temperature. Membranes were then washed three times in TBST and bands were visualized with ECL detection kits (GE Healthcare, RPN2209) according to the manufacturer’s instructions.

### Data analysis

Thermo Scientific Proteome Discoverer software suite 1.4 with the SEQUEST search engine and the mouse FASTA database from UniProt (released on October 16th, 2015) was used to analyze LC-MS/MS data. In the SEQUEST search engine, full trypsin specificity was selected, two missed cleavages were allowed, carbamidomethylation (C) and TMT 6-plex (K and peptide N-terminal) were set as the static modification, oxidation (M) was set as the dynamic modification, precursor ion mass tolerances were set at 20 ppm for all MS data acquired using an Orbitrap mass analyzer, and the fragment ion mass tolerance was set as 20 mmu for all MS/MS spectra acquired. Two or more unique peptides per protein had to be identified to list the protein as a hit. Proteins that scored 10 or more were selected for subsequent bioinformatics analysis. The ratio values of proteins labeled with TMT6-128 and TMT6-127 were adjusted using the beta tubulin ratio value as internal control. The thresholds for downregulation and upregulation were set at 0.5 and 2.0 respectively.

FunRich software (version 2.1.2) (www.funrich.org) was used to classify the proteins using UniProt Database (released on July 21st, 2015). The ClueGO cytoscape plugin (version 2.1.7) was used to analyze the functional interaction networks of the differentially expressed proteins. The REACTOME ontology database (released on May 5th, 2015) was used. Two-sided hypergeometric tests with Benjamini-Hochberg correction method were performed to minimize the false discovery rate. Pathways with *P* value of 0.01 or less were considered as significance. The WebGestalt online toolkit (http://bioinfo.vanderbilt.edu/webgestalt/) was used to run pathway enrichment analysis and the significance level was set at 0.0001. Cytoscape software (version 3.1.1) with Wikipathways plugin was used to visualize the protein-protein interactions matching the Wikipathways database. The mass spectrometry proteomics data have been deposited to the ProteomeXchange Consortium via the PRIDE partner repository with the data identifier PXD001935 [36].

## Additional files

Additional file 1: Table S1. Detailed Information of the Differentially Expressed Proteins. (XLSX 106 kb) Additional file 2: Table S2. WikiPathways Enrichment Analysis of the Differentially Expressed Proteins. (DOCX 17 kb)
