# Supplementary material for: Investigation of proteome changes in osteoclastogenesis in low serum culture system using quantitative proteomics
Source: Proteome Sci. 2016 Mar 31;14:8. doi: 10.1186/s12953-016-0097-6 (PMC4815185; doi:10.1186/s12953-016-0097-6)
Supplement: Additional file 2: Table S2. — WikiPathways Enrichment Analysis of the Differentially Expressed Proteins. (DOCX 17 kb) [file 12953_2016_97_MOESM2_ESM.docx]

**WikiPathways Enrichment Analysis**

| PathwayName | #Gene | EntrezGene | Statistics |
| --- | --- | --- | --- |
| [Electron Transport Chain](http://bioinfo.vanderbilt.edu/webgestalt/draw_wikipathway.php?organism=mmusculus&wp_id=WP295&values=NA,NA,NA,NA,NA,NA,NA,NA,NA,NA,NA,NA,NA,NA,NA,NA,NA,NA,NA,NA,NA,NA,NA,NA,NA,NA,NA,NA,NA,NA,NA,NA,NA,NA,NA,NA,NA,NA,NA,NA,NA,NA,NA,NA,NA&enriched_ids=75406,68375,66043,225887,57423,66416,67680,11958,227197,27425,11739,66108,72900,70316,11957,68194,11946,407785,67003,66046,17709,226646,66945,22273,17992,68342,28080,12858,78330,22272,66414,17995,67130,11947,67273,68349,68202,71679,17993,11740,66694,11949,17991,11950,12857&timestamp=1446275450) | [45](http://bioinfo.vanderbilt.edu/webgestalt/htdocs/final_wiki_geneset_file_1446275450.html#Electron Transport Chain) | 75406 68375 225887 66043 57423 66416 67680 227197 11958 27425 11739 66108 72900 70316 11957 68194 407785 11946 6604667003 226646 17709 17992 22273 66945 68342 28080 12858 78330 22272 66414 17995 67130 11947 67273 68349 68202 7167966694 11740 17993 11949 17991 11950 12857 | C=102;O=45;E=0.92;R=48.90;rawP=1.75e-64;adjP=9.80e-63 |
| [Oxidative phosphorylation](http://bioinfo.vanderbilt.edu/webgestalt/draw_wikipathway.php?organism=mmusculus&wp_id=WP1248&values=NA,NA,NA,NA,NA,NA,NA,NA,NA,NA,NA,NA,NA,NA,NA,NA,NA,NA,NA,NA,NA,NA,NA,NA,NA,NA,NA,NA,NA,NA,NA,NA&enriched_ids=68342,75406,28080,78330,68375,66043,225887,57423,17995,66416,11958,227197,67130,27425,54411,11947,67273,68349,68202,70495,71679,66108,17993,72900,11957,407785,11946,17991,66046,11950,226646,17992&timestamp=1446275450) | [32](http://bioinfo.vanderbilt.edu/webgestalt/htdocs/final_wiki_geneset_file_1446275450.html#Oxidative phosphorylation) | 75406 68375 225887 66043 57423 66416 227197 11958 27425 70495 66108 72900 11957 11946 407785 66046 226646 1799228080 68342 78330 17995 67130 54411 11947 67273 68349 68202 71679 17993 17991 11950 | C=60;O=32;E=0.54;R=59.12;rawP=1.17e-49;adjP=3.28e-48 |
| [Amino Acid metabolism](http://bioinfo.vanderbilt.edu/webgestalt/draw_wikipathway.php?organism=mmusculus&wp_id=WP662&values=NA,NA,NA,NA,NA,NA,NA,NA,NA,NA,NA,NA,NA,NA,NA,NA,NA,NA,NA,NA,NA,NA,NA,NA,NA,NA,NA,NA&enriched_ids=108682,11992,18263,107869,227095,14645,56454,18597,15356,14661,15107,13382,12974,14719,17448,14194,78920,72039,18293,17850,11364,56451,27402,102436,14660,58875,11429,66945&timestamp=1446275450) | [28](http://bioinfo.vanderbilt.edu/webgestalt/htdocs/final_wiki_geneset_file_1446275450.html#Amino Acid metabolism) | 108682 227095 15356 14661 13382 17448 14719 78920 14194 18293 72039 14660 11429 66945 18263 11992 107869 14645 1859756454 15107 12974 11364 17850 27402 56451 102436 58875 | C=112;O=28;E=1.01;R=27.71;rawP=2.63e-32;adjP=4.91e-31 |
| [TCA Cycle](http://bioinfo.vanderbilt.edu/webgestalt/draw_wikipathway.php?organism=mmusculus&wp_id=WP434&values=NA,NA,NA,NA,NA,NA,NA,NA,NA,NA,NA,NA,NA,NA,NA,NA,NA,NA,NA&enriched_ids=20916,20917,68263,67680,18597,67834,13382,17448,12974,14194,78920,18293,269951,27402,56451,15929,11429,235339,66945&timestamp=1446275450) | [19](http://bioinfo.vanderbilt.edu/webgestalt/htdocs/final_wiki_geneset_file_1446275450.html#TCA Cycle) | 20916 67680 67834 13382 17448 78920 14194 18293 269951 235339 11429 66945 20917 68263 18597 12974 56451 27402 15929 | C=32;O=19;E=0.29;R=65.81;rawP=3.17e-31;adjP=4.44e-30 |
| [Fatty Acid Beta Oxidation](http://bioinfo.vanderbilt.edu/webgestalt/draw_wikipathway.php?organism=mmusculus&wp_id=WP1269&values=NA,NA,NA,NA,NA,NA,NA,NA,NA,NA,NA,NA,NA,NA,NA&enriched_ids=11370,433256,14571,11364,110446,93747,231086,11409,270076,12908,97212,15107,12896,13382,74205&timestamp=1446275450) | [15](http://bioinfo.vanderbilt.edu/webgestalt/htdocs/final_wiki_geneset_file_1446275450.html#Fatty Acid Beta Oxidation) | 11370 12908 270076 12896 13382 231086 110446 11409 15107 14571 433256 11364 93747 97212 74205 | C=69;O=15;E=0.62;R=24.10;rawP=6.34e-17;adjP=7.10e-16 |
| [Mitochondrial LC-Fatty Acid Beta-Oxidation](http://bioinfo.vanderbilt.edu/webgestalt/draw_wikipathway.php?organism=mmusculus&wp_id=WP401&values=NA,NA,NA,NA,NA,NA,NA&enriched_ids=11409,11370,97212,15107,11364,12896,74205&timestamp=1446275450) | [7](http://bioinfo.vanderbilt.edu/webgestalt/htdocs/final_wiki_geneset_file_1446275450.html#Mitochondrial LC-Fatty Acid Beta-Oxidation) | 11370 12896 11409 15107 11364 97212 74205 | C=16;O=7;E=0.14;R=48.49;rawP=4.98e-11;adjP=4.65e-10 |
| [Fatty Acid Biosynthesis](http://bioinfo.vanderbilt.edu/webgestalt/draw_wikipathway.php?organism=mmusculus&wp_id=WP336&values=NA,NA,NA,NA,NA,NA,NA,NA&enriched_ids=67856,51798,433256,93747,26922,52538,15107,74205&timestamp=1446275450) | [8](http://bioinfo.vanderbilt.edu/webgestalt/htdocs/final_wiki_geneset_file_1446275450.html#Fatty Acid Biosynthesis) | 52538 51798 26922 15107 67856 433256 93747 74205 | C=29;O=8;E=0.26;R=30.58;rawP=1.51e-10;adjP=1.21e-09 |
| [Synthesis and Degradation of Ketone Bodies](http://bioinfo.vanderbilt.edu/webgestalt/draw_wikipathway.php?organism=mmusculus&wp_id=WP543&values=NA,NA,NA,NA&enriched_ids=67041,15356,71911,110446&timestamp=1446275450) | [4](http://bioinfo.vanderbilt.edu/webgestalt/htdocs/final_wiki_geneset_file_1446275450.html#Synthesis and Degradation of Ketone Bodies) | 71911 15356 110446 67041 | C=5;O=4;E=0.05;R=88.68;rawP=3.25e-08;adjP=2.27e-07 |
| [Glycolysis and Gluconeogenesis](http://bioinfo.vanderbilt.edu/webgestalt/draw_wikipathway.php?organism=mmusculus&wp_id=WP157&values=NA,NA,NA,NA,NA,NA,NA&enriched_ids=27402,17448,14719,18597,68263,235339,13382&timestamp=1446275450) | [7](http://bioinfo.vanderbilt.edu/webgestalt/htdocs/final_wiki_geneset_file_1446275450.html#Glycolysis and Gluconeogenesis) | 13382 14719 17448 235339 68263 18597 27402 | C=51;O=7;E=0.46;R=15.21;rawP=3.84e-07;adjP=2.39e-06 |
| [Urea cycle and metabolism of amino groups](http://bioinfo.vanderbilt.edu/webgestalt/draw_wikipathway.php?organism=mmusculus&wp_id=WP426&values=NA,NA,NA,NA,NA&enriched_ids=56454,18263,12715,14661,12709&timestamp=1446275450) | [5](http://bioinfo.vanderbilt.edu/webgestalt/htdocs/final_wiki_geneset_file_1446275450.html#Urea cycle and metabolism of amino groups) | 12709 14661 12715 18263 56454 | C=22;O=5;E=0.20;R=25.19;rawP=1.36e-06;adjP=7.62e-06 |
| [Tryptophan metabolism](http://bioinfo.vanderbilt.edu/webgestalt/draw_wikipathway.php?organism=mmusculus&wp_id=WP79&values=NA,NA,NA,NA,NA,NA,NA&enriched_ids=11669,270076,15107,18293,110446,11671,93747&timestamp=1446275450) | [7](http://bioinfo.vanderbilt.edu/webgestalt/htdocs/final_wiki_geneset_file_1446275450.html#Tryptophan metabolism) | 270076 18293 110446 15107 11669 93747 11671 | C=67;O=7;E=0.60;R=11.58;rawP=2.55e-06;adjP=1.30e-05 |

•C: the number of reference genes in the category

•O: the number of genes in the gene set and also in the category

•E: the expected number in the category

•R: ratio of enrichment

•rawP: P-value from hypergeometric test

•adjP: P-value adjusted by the multiple test adjustment
